# A novel serum calprotectin (MRP8/14) particle-enhanced immuno-turbidimetric assay (sCAL turbo) helps to differentiate systemic juvenile idiopathic arthritis from other diseases in routine clinical laboratory settings

**DOI:** 10.1186/s40348-023-00168-0

**Published:** 2023-10-25

**Authors:** Dirk Foell, Melanie Saers, Carolin Park, Ninna Brix, Mia Glerup, Christoph Kessel, Helmut Wittkowski, Claas Hinze, Lillemor Berntson, Anders Fasth, Charlotte Myrup, Ellen Nordal, Marite Rygg, Henrik Hasle, Birgitte Klug Albertsen, Troels Herlin, Dirk Holzinger, Christian Niederberger, Bernhard Schlüter

**Affiliations:** 1 Department of Pediatric Rheumatology and Immunology, University Children’s Hospital Muenster, Muenster, D-48149 Germany; 2 Department of Pediatric and Adolescent Medicine, Aalborg University Hospital, Aalborg, Denmark; 3 Department of Pediatrics and Adolescent Medicine, Aarhus University Hospital, Aarhus, Denmark; 4 Department of Women’s and Children’s Health, Uppsala University, Uppsala, Sweden; 5 Department of Pediatrics, Institute of Clinical Sciences, Sahlgrenska Academy, University of Gothenburg, Gothenburg, Sweden; 6 Department of Pediatrics, Copenhagen University Hospital, Copenhagen, Denmark; 7 Department of Pediatrics, University Hospital of North Norway, Tromso, Norway; 8 Department of Clinical Medicine, Arctic University of Norway, Tromso, Norway; 9 Department of Pediatrics, St. Olavs Hospital, Trondheim, Norway; 10 Department of Clinical and Molecular Medicine, Norwegian University of Science and Technology, Trondheim, Norway; 11 Department of Pediatric Hematology-Oncology, University of Duisburg-Essen, Essen, Germany; 12 Department of Applied Health Sciences, University of Applied Sciences Bochum, Bochum, Germany; 13 BÜHLMANN Laboratories AG, Schönenbuch, Switzerland; 14 Central Laboratory, University Hospital Muenster, Muenster, Germany

**Keywords:** Biomarkers, Fever of unknown origin, Still’s syndrome, Systemic inflammation

## Abstract

**Background:**

Differential diagnosis in children with signs of unprovoked inflammation can be challenging. In particular, differentiating systemic juvenile idiopathic arthritis (SJIA) from other diagnoses is difficult. We have recently validated the complex of myeloid-related proteins 8/14 (MRP8/14, also known as S100A8/A9 complex or serum calprotectin) as a helpful biomarker supporting the diagnosis of SJIA. The results were subsequently confirmed with a commercial ELISA. However, further optimization of the analytical technology is important to ensure its feasibility for large-scale use in routine laboratory settings.

**Methods:**

To evaluate the accuracy in identifying children with SJIA, the performance of a particle-enhanced immuno-turbidimetric assay for serum calprotectin (sCAL turbo) on an automated laboratory instrument was analyzed. Samples from 615 children were available with the diagnoses SJIA (*n* = 99), non-systemic JIA (*n* = 169), infections (*n* = 51), other inflammatory diseases (*n* = 126), and acute lymphoblastic leukemia (ALL, *n* = 147). In addition, samples from 23 healthy controls were included.

**Results:**

The sCAL turbo assay correlated well with the MRP8/14 ELISA used in previous validation studies (*r* = 0.99, *p* < 0.001). It could reliably differentiate SJIA from all other diagnoses with significant accuracy (cutoff at 10,500 ng/ml, sensitivity 84%, specificity 94%, ROC area under curve 0.960, *p* < 0.001).

**Conclusions:**

Serum calprotectin analyses are a helpful tool supporting the diagnosis of SJIA in children with prolonged fever or inflammatory disease. Here, we show that an immuno-turbidimetric assay for detection of serum calprotectin on an automated laboratory instrument can be implemented in clinical laboratory settings to facilitate its use as a diagnostic routine test in clinical practice.

**Supplementary Information:**

The online version contains supplementary material available at 10.1186/s40348-023-00168-0.

## Background

Systemic juvenile idiopathic arthritis (SJIA) is a serious disease in children with prominent systemic inflammatory features, which can be accompanied by arthritis. The International League of Associations for Rheumatology (ILAR) classification criteria still consider SJIA a category of juvenile idiopathic arthritis (JIA) [1]. Phenotypically, it is characterized by fever, an evanescent rash, serositis, reticuloendothelial involvement, and articular manifestations. Today, SJIA is seen as part of the autoinflammatory disease spectrum [2]. In the initial phase, sterile hyperinflammation is driven by innate immune processes with a prominent role of cytokines such as interleukin (IL)-1 and IL-6. If not stopped during this phase, the disease can progress into a later phase involving complex immune dysregulation also leading to a destructive chronic arthritis [3, 4]. In addition, patients with SJIA are prone to complications, including life-threatening hyperinflammation (macrophage activation syndrome, SJIA-MAS) and the more recently described SJIA-associated lung disease (SJIA-LD) [5]. Meanwhile, the treatment arsenal in SJIA has expanded markedly, and state-of-the-art therapeutic approaches include approved biologics targeting IL-1 and IL-6 pathways.

It has been well-documented that starting treatment as early as possible is favorable for the effectiveness of cytokine blockade. Early intervention could potentially prevent the biphasic course with the threatening development of chronic inflammatory processes [6–9]. Nevertheless, many patients are still treated too late or not successfully because the diagnosis is not made in the optimal time window (“window of opportunity”). At onset, patients frequently present with fever of unknown origin and/or with nonspecific signs and symptoms. In this initial phase, and particularly in the absence of arthritis, distinguishing SJIA from other diagnostic alternatives remains highly challenging. Therefore, SJIA is still seen as a diagnosis of exclusion, which can significantly delay final diagnosis and initiation of adequate treatment [10, 11]. To enable inclusion of patients without arthritis into clinical practice recommendations, treatment plans have been developed for “classic” (ILAR defined) SJIA and “probable” SJIA without arthritis [11]. Consensus treatment plans developed by the Childhood Arthritis and Rheumatology Research Alliance (CARRA) included a case definition that requires arthritis of any duration, rather than the 6 weeks required per the ILAR criteria [12]. New expert initiatives are currently working on revised classification criteria [2]. An ongoing European League of Associations for Rheumatology (EULAR)/Paediatric Rheumatology European Society (PReS) task force reached consensus for a novel concept of Still’s disease as one continuum, including both SJIA and its counterpart with onset in adulthood termed adult-onset Still’s disease (AOSD).

Biomarkers differentiating SJIA in its initial inflammatory phase from the main differential diagnoses, i.e., infections or malignancies, may support early diagnosis and thus a timely start of therapy. Among published biomarkers differentiating SJIA from infections, the heterocomplex S100A8/A9 appears especially promising [13, 14]. This complex consists of two members of the S100 family of calcium-binding proteins, which are expressed and released by cells of myeloid origin. The proteins are also referred to as myeloid-related proteins 8 and 14, and the complex has been termed MRP8/14 or “serum calprotectin” [15]. As danger signals they are secreted by activated myeloid cells, phagocyte-specific S100 proteins amplify disease processes in SJIA by activating Toll-like receptor 4. Serum levels are extremely elevated in active SJIA, and the measurement of S100A8/A9 can be used for the diagnosis of SJIA [16–18]. These results were recently validated with a commercially available ELISA [19].

However, the analytical technique needs further optimization to ensure easy availability and enable large-scale use in routine laboratories. To achieve this objective, we collaborated with the central laboratory at our university hospital to establish an immuno-turbidimetric assay for the measurement of serum calprotectin in serum samples on an automated laboratory instrument. In addition, the inclusion of more patients with hematological malignancies was warranted, as especially leukemias can cause fever and joint pain at initial presentation in children [20].

## Patients and methods

### Patients

We included patients with fever of unknown origin, signs of unprovoked inflammation, or relevant differential diagnoses of Still’s disease, who were undergoing a diagnostic checkup for suspected inflammatory or malignant diseases. By definition, there was no definite SJIA diagnosis in any of these patients at the time of sampling. In these patients, the diagnosis used in the statistical analyses was confirmed post hoc by the referring physicians as previously reported [19]. The later confirmed diagnoses of the patients were SJIA (*n* = 99), nonsystemic JIA (nsJIA, *n* = 169), systemic febrile infections (*n* = 51), other systemic inflammatory diseases (SIDs, *n* = 126), and acute lymphoblastic leukemia (ALL, *n* = 147). The patients with fever of unknown origin were collected at the University of Muenster as reported before [19]. The NOPHO Leukemia BioBank (NLBB) in Uppsala, Sweden, provided the serum samples of consecutive ALL patients from Aalborg and Aarhus University Hospitals, Denmark [21]. Patients with nsJIA were from the Nordic JIA cohort as previously described in detail [22, 23]. In addition, samples from 23 healthy controls were included at the University of Muenster. They were from children who had presented to the pediatric endocrinology unit with concern for short or tall stature but for whom an inflammatory condition had been ruled out. Thus, in total, 615 samples were available for analysis.

### Sample handling

Calprotectin is expressed and stored in huge amounts in leukocytes. To avoid unspecific release of the biomarker into collected samples, centrifugation and separation within 4 h after blood drawing were performed. Serum supernatants were stored at − 80 °C until processing. Calprotectin complexes are stable for at least 3 days at room and for months below − 20 °C, even with freeze/thaw cycles 3 times or more [19].

### Biomarker analyses

To evaluate the accuracy in identifying children with SJIA, the performance of an immuno-turbidimetric assay for measurements of serum calprotectin (BÜHLMANN sCAL® turbo) implemented into the automated laboratory instrument (Roche cobas® c502) at the central clinical laboratory of the University Hospital Muenster. The BÜHLMANN sCAL turbo test is a particle-enhanced immuno-turbidimetric assay (PETIA) with a detection range of 230–15,000 ng/ml (extended range up to 225,000 ng/ml by dilution of 1:15) in sample volumes of only 2–3 µl. The upper limit of serum levels in 160 healthy adults was 2740 ng/ml (90% confidence interval), according to the manufacturer’s information. In addition, an established MRP8/14 ELISA from BÜHLMANN (EK-MRP8/14) was used according to the manufacturer’s protocol to compare formerly reported values to the results obtained with the new immuno-turbidimetric assays. Samples were diluted 1:100 initially; for results below or above the assay range, a lower or higher dilution was applied.

### Statistics

The statistical analysis of rank differences was performed using two-sided ANOVA and the Kruskal–Wallis test with Dunn’s correction for multiple comparisons. Receiver operating characteristic (ROC) curves were plotted to determine the accuracy of inflammation marker measurements as a diagnostic test and for calculation of cut-off values. The performance of the models was evaluated using the area under the receiver operating characteristic curve (AUC), sensitivity, specificity, positive or negative likelihood ratio, and accuracy using a threshold of *p* < 0.05. Spearman’s *p* correlations were calculated. SPSS 25.0 for Microsoft Windows (SPSS, Chicago, IL, USA) and GraphPad Prism version 8.0 for Mac (GraphPad Software, La Jolla, CA, USA) were used for statistical analyses. Unless stated otherwise, data are expressed as mean with 95% CI.

## Results

### Comparison of patient characteristics

Healthy controls were significantly older than patients from all disease groups. SJIA patients were characterized by high WBC counts, ESR, and CRP, but notably, these parameters were not significantly elevated compared to infections as a major differential diagnosis. CRP was higher in SJIA than in ALL, which is another main differential diagnosis in patients with fever of unknown origin or signs of unprovoked inflammation. ALL patients also had abnormalities that would not be typically seen in SJIA, such as markedly elevated LDH or reduced platelet counts. Basic characteristics of the patients are summarized in Table 1. Further details are provided in Supplemental Table 1.

**Table 1:** Characteristics of the patients with available serum samples

	**SJIA**	**Infections**	**SIDs**	**nsJIA**	**ALL**	**Controls**
**Samples, *n***	99	51	126	169	147	23
**Age**	6.8 (0.6–16.5)	4.5 (0.3–16.9)	5.5 (0.4–17.7)	6.4 (0.2–15.8)	4.4 (0.5–18.8)	13.2 (4.8–16.9)
**Sex, f/m (% female)**	45/49 (45)	31/20 (61)	60/66 (48)	114/55 (67)	67/75 (46)	11/12 (48)
**WBC, cells/µl**	14,700§ (3700–40,900)	11,375 (3400–38,000)	9520 (1100–25,000)	9560 (6700–22,100)	8700 (300–726,000)	n.d
**ESR, mm/h**	83 (4–170)	65 (5–109)	27# (1–140)	10## (1–104)	61 (9–160)	n.d
**CRP, mg/dl**	9.5* (1.2–41.2)	8.0** (1.2–44.1)	4.3 (1.0–32.8)	3.8 (0.1–8.9)	2.0 (0.8–18.5)	< 0.5

All data expressed as median (range) except otherwise stated

*WBC* White blood cells, *ESR* Erythrocyte sedimentation rate, *CRP* C-reactive protein, *n.d *Not determined

§WBC count significantly higher in SJIA compared to SIDs (*p* < 0.001) and ALL (*p* = 0.005)

#ESR significantly lower in SIDs compared to SJIA (*p* < 0.001), infections (*p* < 0.01), and ALL (*p* < 0.001)

##ESR significantly lower in nsJIA compared all other disease groups (*p* < 0.001)

^*^CRP significantly higher in SJIA compared to SIDs, nsJIA, and ALL (*p* < 0.001 for all)

^**^CRP significantly higher in infections compared to ALL (*p* < 0.001)

### Correlation between sCAL turbo and MRP8/14 ELISA

**Fig. 1:**
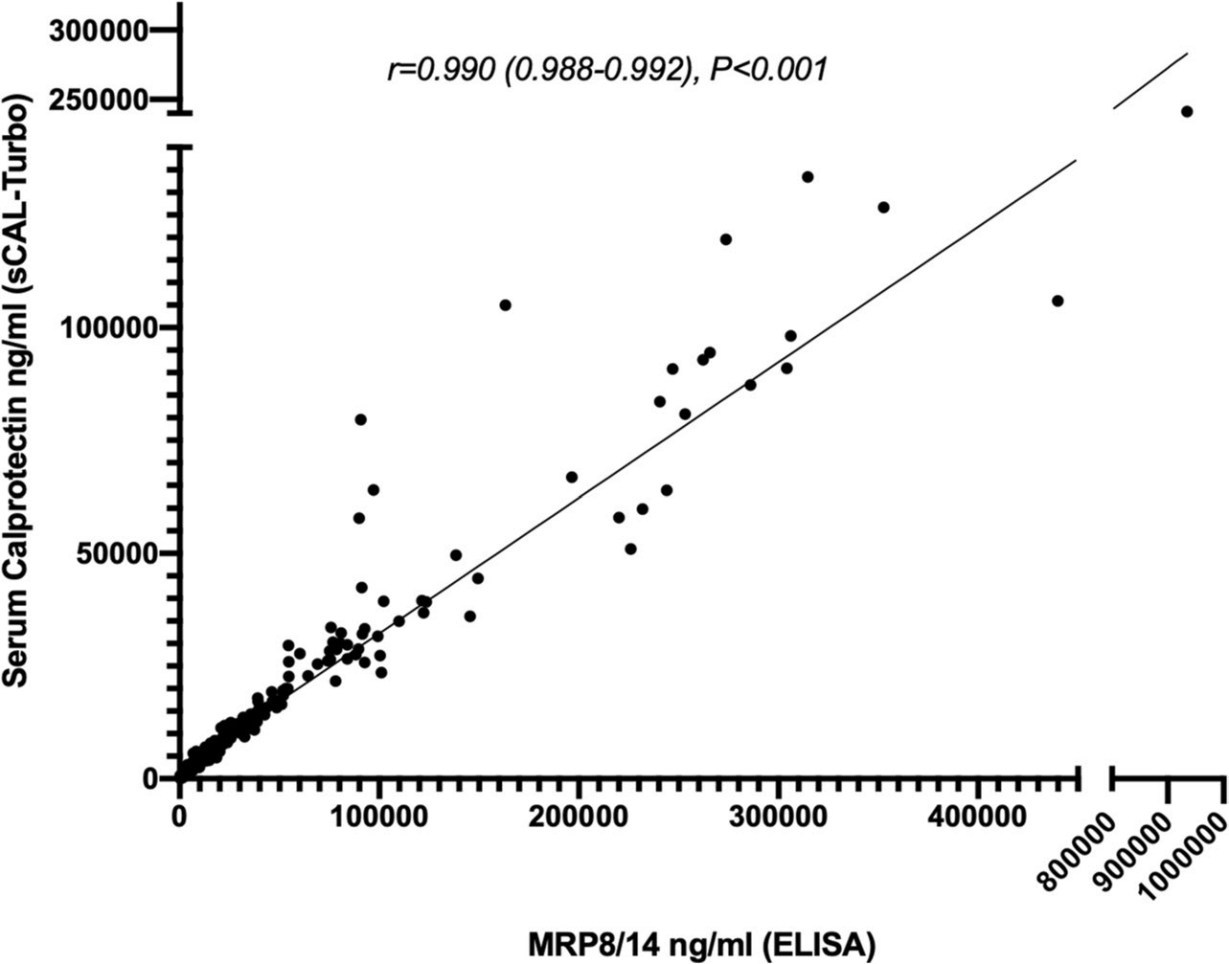
Correlation between sCAL turbo and MRP8/14 ELISA. Spearman’s *p* correlations were calculated in 299 samples with parallel analysis of the S100A8/A9 serum concentration by either the sCAL turbo or the MRP8/14 ELISA (both from BÜHLMANN)

We first analyzed how levels established with the new sCAL turbo assay would compare to levels determined by the MRP8/14 ELISA that was used in previous validation studies (Fig. 1). In total, 299 samples (from all disease groups except nsJIA and ALL) were run on both assays, and respective data were used for comparison and correlation analyses. We found an excellent correlation of sCAL turbo values to those produced by the ELISA (*r* = 0.99, *p* < 0.001). Values generated with the MRP8/14 ELISA were higher than those determined by the sCAL turbo assay (mean 2.5-fold difference).

### Levels of serum calprotectin in different disease groups

**Fig. 2:**
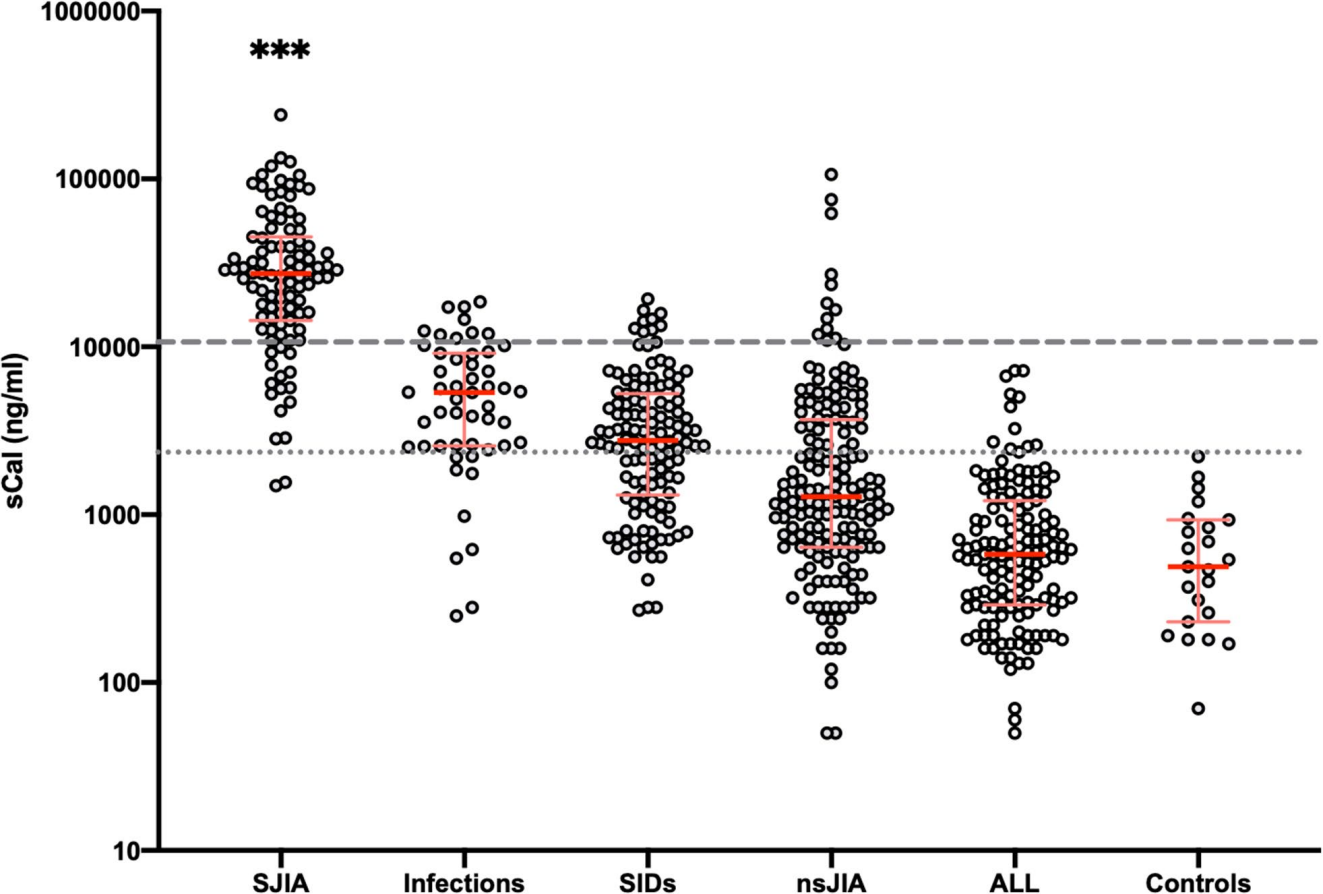
Results of sCAL turbo measurements in different groups of patients. Results of sCAL measurements in patient groups are shown using individual scatter plots, with red line showing median, error bars showing interquartile range (logarithmic scale of *y*-axis). The dotted gray line shows the upper limit of healthy controls. The dashed gray line shows the cutoff at 10,500 ng/ml which revealed the best accuracy in differentiating SJIA from other differential diagnoses (as determined by ROC analyses, see below). ***p < 0.001 for SJIA vs. all other groups (Kruskal-Wallis test with Dunn’s correction for multiple comparison)

Overall, the sCAL turbo assay revealed an excellent performance. In healthy children, all measured values remained below the upper limit of healthy controls as provided by the manufacturer, with the highest level measured at 2220 ng/ml. The concentrations in samples from all patient groups with diverse systemic inflammatory diseases were elevated above the maximal levels of healthy controls as quantified in this study (in the SJIA group 98% of measurements, in the infection group 84%, and in the SIDs group 62%). In contrast, only 34% of patients with nsJIA revealed values above the upper limit of normal, while only few patients with ALL (8%) presented with values of such level. There were markedly different sCAL levels between the patient groups, with those in SJIA being significantly higher compared to all others, thus including the major differential diagnoses in FUO (i.e., infections, SIDs, and leukemia) (Fig. 2 and Supplemental Table 2).

**Table 2:** Accuracy (ROC analyses) of sCAL turbo measurements in differentiating groups

	***SJIA vs all groups***	***SJIA vs infections***	***SJIA vs ALL***
***AUC (95%CI)***	0.960 (0.941–0.978)	0.908 (0.862–0.953)	0.992 (0.985–0.999)
***Cutoff (ng/ml)***	10,500	10,500	10,500
***Sensitivity (%)***	84	84	84
***Specificity (%)***	94	82	100
***Positive likelihood ratio (LR* +*)***	14.0	4.7	$\infty$
***Negative likelihood ratio (LR −)***	0.17	0.20	0.16

### Assay performance in differentiating SJIA from other diagnoses

**Fig. 3:**
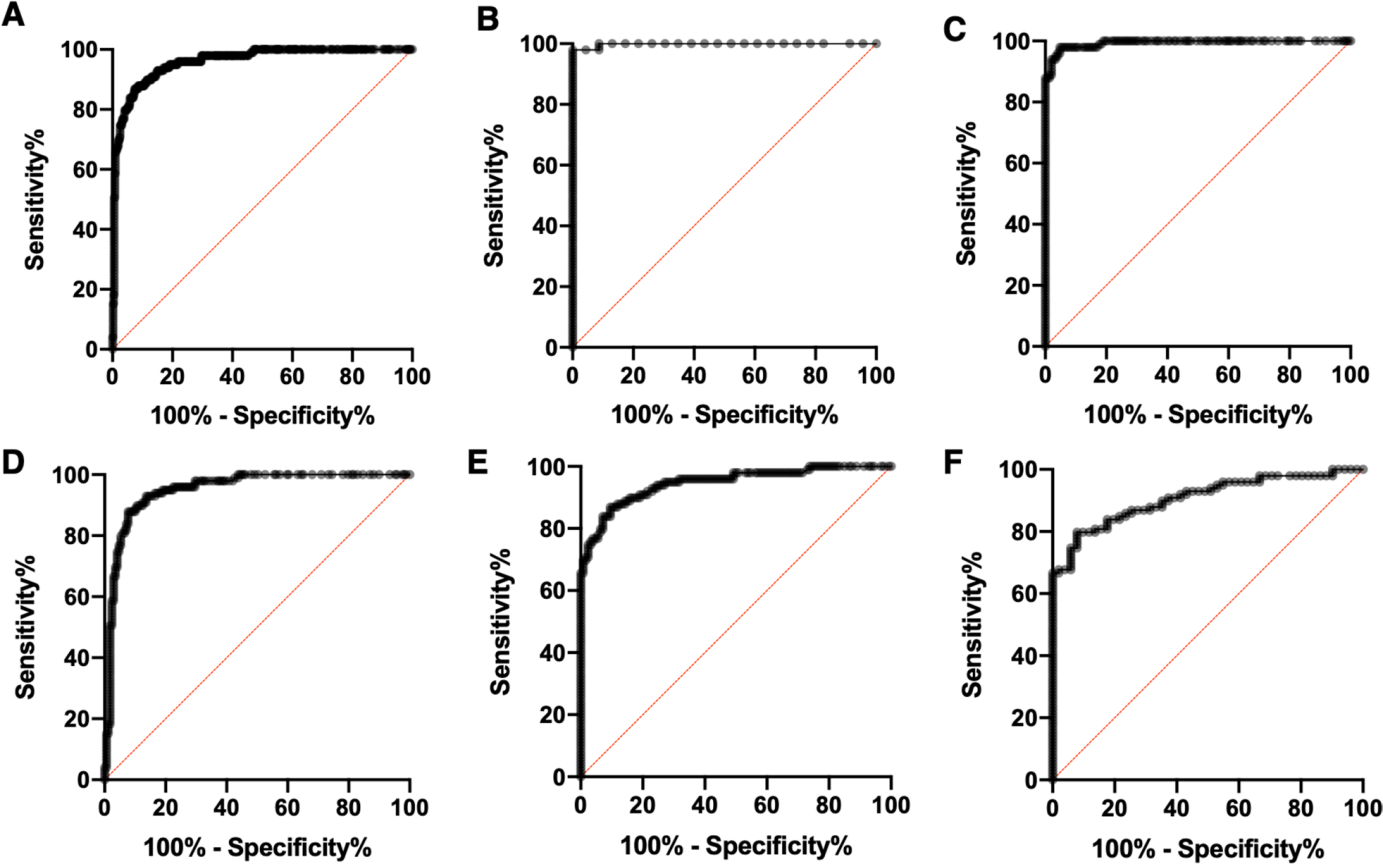
ROC curves of sCAL turbo analyses. ROC analyses were performed to determine the overall accuracy of sCAL measurements in differentiating patient groups. The ROC curves show analyses for **A** SJIA vs all other patients (*AUC* = 0.960, *p* < 0.001), **B** SJIA vs healthy controls (*AUC* = 0.998, *p* < 0.001), **C** SJIA vs ALL (*AUC* = 0.993, *p* < 0.001), **D** SJIA vs nsJIA (*AUC* = 0.951, *p* < 0.001), **E** SJIA vs SIDs (*AUC* = 0.946, *p* < 0.001), and **F** SJIA vs infections (*AUC* = 0.908, *p* < 0.001)

**Fig. 4:**
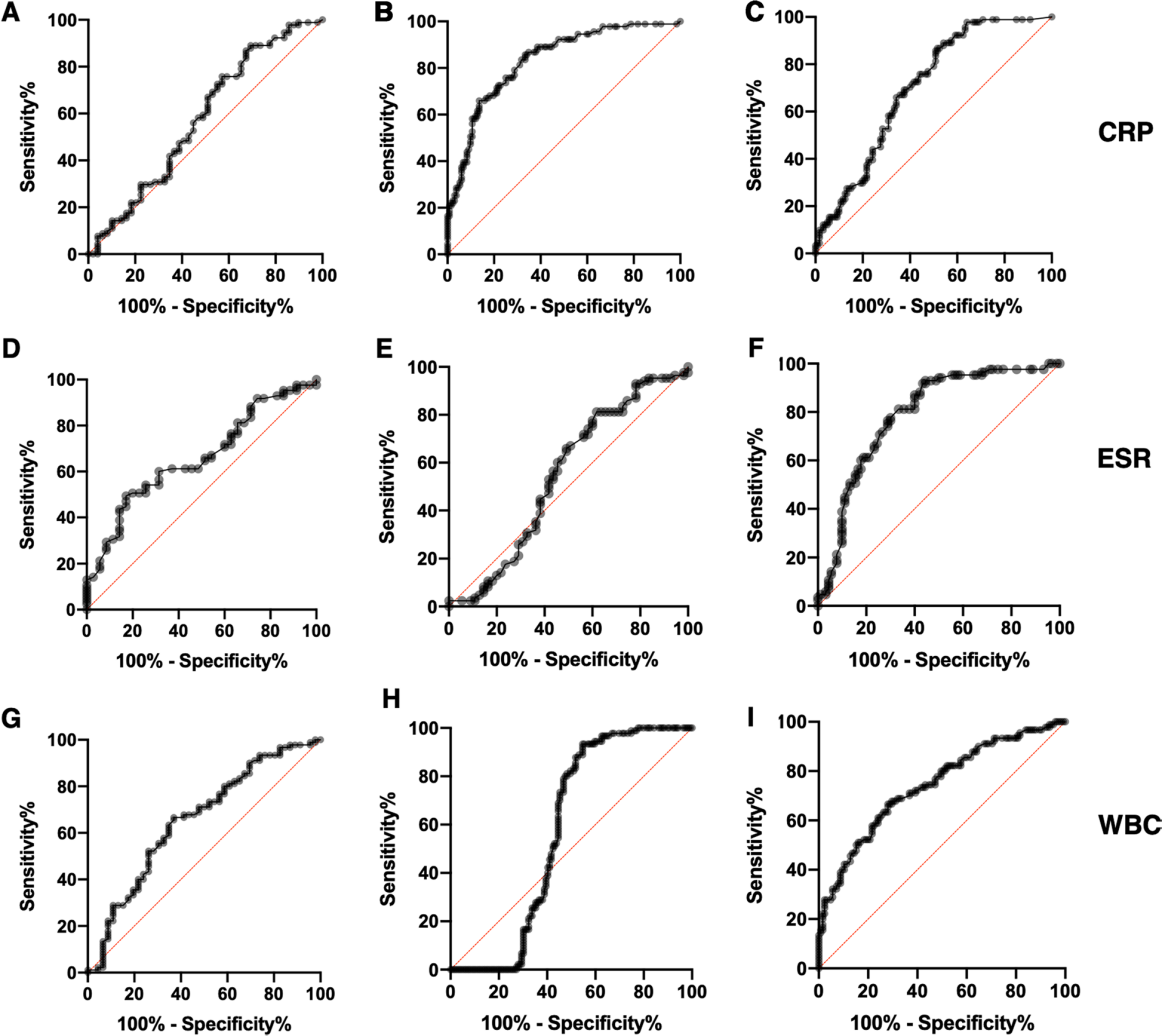
ROC curves of routine inflammatory marker analyses. ROC analyses were performed to determine the overall accuracy of CRP, ESR, and WBC measurements in differentiating the major differential diagnoses in FUO, i.e., SJIA versus infections and leukemia. The ROC curves show analyses for **A** CRP in SJIA vs infections (*AUC* = 0.577, *p* = 0.134), **B** CRP in SJIA vs ALL (*AUC* = 0.831, *p* < 0.001), **C** CRP in SJIA vs SIDs (*AUC* = 0.702, *p* < 0.001), **D** ESR in SJIA vs infections (*AUC* = 0.658, *p* = 0.007), **E** ESR in SJIA vs ALL (0.547, *p* = 0.353), **F** ESR in SJIA vs SIDs (*AUC* = 0.789, *p* < 0.001), **G** WBCs in SJIA vs infections (*AUC* = 0.656, *p* = 0.003), **H** WBCs in SJIA vs ALL (*AUC* = 0.575, *p* = 0.056), **I** WBCs in SJIA vs SIDs (*AUC* = 0.741, *p* < 0.001). *FUO*, fever of unknown origin; *SID*, systemic inflammatory diseases

The sCAL turbo assay could reliably differentiate SJIA from all other diagnoses with significant accuracy (cutoff at 10,500 ng/ml, sensitivity 84%, specificity 94%, ROC AUC 0.960, *p* < 0.001) (Table 2). In the SJIA group, 84% of the determined values remained above this threshold, which was the case for 18% of values in the infection, 8% in the SIDs, and 7% in the nsJIA group. The highest quantified sCAL concentration among ALL patients was 7210 ng/ml and thus remained clearly below the determined optimized cutoff for SJIA. Consequently, if sCAL measured in a patient is higher than 10,500 ng/ml, it is very likely that SJIA is the underlying cause of FUO (LR + , positive likelihood ratio 14.0 versus all other groups). Vice versa, it is unlikely to falsely diagnose SJIA in this scenario (LR − , negative likelihood ratio of 0.17 and 0.20 versus all other groups and versus infection, respectively). In case of higher levels, it is even more likely that SJIA is the correct diagnosis. Using a stricter cutoff at 15,000 ng/ml, the LR + increases to 37.5 (versus all groups) and to 12.5 versus infections (Supplemental Table 3). The ROC curves showing the accuracy in differentiating the individual patient groups are shown in Fig. 3. In contrast, CRP, ESR, or WBCs allowed no robust differentiation of SJIA from neither of the three major differential diagnoses in suspected SJIA, i.e., infections, SIDs, and leukemia (Fig. 4).

## Discussion

Here, we show that an immuno-turbidimetric assay for detection of serum calprotectin on an automated laboratory instrument can be implemented in clinical laboratory settings to facilitate its use as a diagnostic routine test in clinical practice. The sCAL turbo assay on an automated instrument can provide results within 1 h. The combination of an automated process with less labor burden and time consumption also helps to limit resources. In a realistic estimation, the future cost efficiency could be comparable to other routine laboratory parameters. Of note, values generated with the formerly used MRP8/14 ELISA are higher than with the sCAL turbo assay (mean 2.5-fold difference). This has to be taken into account when comparing present values to those of previous studies. The mean values and cutoffs separating SJIA from infections or other diseases are now lower than reported for the commercial MRP8/14 ELISA [19] but remain comparable to previous data demonstrating performance of an experimental ELISA system [24, 25].

In the initial phase of disease when arthritis is absent, SJIA presents with nonspecific signs and symptoms (fever, rash, arthralgia, elevation of inflammatory markers), which can render definite diagnosis complicated and potentially delay targeted treatment [11]. Therefore, an early diagnosis is often challenging since many differential diagnoses need to be considered, including infections, malignancy, and other autoinflammatory or autoimmune diseases [19, 26, 27]. Biomarkers capable of distinguishing SJIA from its differential diagnoses can aid in this process if they are applicable in routine clinical practice, reproducible with high sensitivity and specificity, and ideally have a clear link to disease pathogenesis [13].

Up to date, the alarmins S100A8 and S100A9 (as complex also called myeloid-related protein 8 and 14, MRP8/14 or known as serum calprotectin) provide the only validated biomarker robustly discriminating between SJIA from differential diagnoses in routine clinical settings [19]. In contrast to other biomarker candidates, serum calprotectin analyses have been validated as a helpful tool supporting the diagnosis of SJIA in children with prolonged fever or inflammatory disease [20]. While our university hospital previously offered this test to centers within Germany, the aim is now to broaden access and establish uniform quantification standards.

Our study has relevant limitations. Firstly, SJIA and other included diagnoses are rather rare diseases; thus, a newly designed prospective study with prespecified aim to test the performance of various biomarkers in FUO was not achievable. Therefore, we rely on a collection of samples used in previous biomarker studies and thus need to accept inhomogeneous characteristics of the patient groups. However, all samples come from carefully designed biorepositories. Age and gender do not impact the sCAL results, at least not beyond infancy. Sample handling errors are very unlikely, and the stability of the analyzed protein complex also facilitates the post hoc use as in the present study. As another limitation, while the collection of FUO patients was prospective and already used for the validation of the MRP8/14 ELISA, the samples from nsJIA and ALL patients were included retrospectively. Those were run on the new sCAL turbo assay exclusively. Thus, a comparison with historic biomarker results was not possible for these samples. Lastly, we were able to include patient samples from the most important differential diagnoses of SJIA, but it was not feasible to include all possible causes of FUO and/or unclear signs of inflammation. It was demonstrated that S100 proteins differentiate well between SJIA even from many other hyperinflammatory conditions [28], but undoubtedly, further conditions including other malignancies than ALL, specific infections not represented by our collection, or further systemic inflammatory diseases such as multisystem inflammatory syndrome in children (MIS-C) may also mimic SJIA. This needs to be considered when interpreting our results. However, sCAL can specifically help to exclude ALL as a differential diagnosis, also due to the fact that neutrophil activity leading to its release into the serum is much lower in ALL, especially in cases with lower neutrophil counts (Supplemental Figs. 1 and 2).

In an individual patient with fever of unknown origin or unprovoked signs of inflammation, the differential diagnosis is impacted by a combination of medical history, clinical symptoms, laboratory features, and imaging findings. Depending on the level of suspicion, the demand for an exclusion of differential diagnoses with near certainty may vary before a definite diagnosis of SJIA is made. This warrants careful considerations, and the diagnostic process will always rely on an evaluation made with caution. While sCAL turbo levels above 10,500 ng/ml put SJIA on the top of the differential diagnoses list (with a specificity against infection of 82% and against ALL of 100%), other conditions cannot be ruled out completely. If the sCAL turbo levels are higher than 15,000 ng/ml, SJIA becomes even more likely (specificity ruling out infections 94%, ruling out ALL 100%), and in cases above 20,000 ng/ml, infections or leukemias can be assumed as highly unlikely. In clinical practice, such a gradual interpretation of sCAL turbo results in combination with other signs and symptoms can steer the workup and facilitate therapeutic decisions.

## Conclusions

An immunoturbidimetric assay for serum-calprotectin (MRP8/14, S100A8/A9) analyses could be demonstrated as a helpful tool supporting the diagnosis of SJIA in children with prolonged fever or inflammatory disease. The implications are profound as making the correct diagnosis of SJIA can be challenging. Our findings provide evidence that S100A8/A9 are not only mechanistic biomarkers that help to distinguish SJIA from its differential diagnoses with high sensitivity and specificity but moreover can be analyzed in a routine laboratory using the sCAL turbo assay on automated standard instruments. In clinical practice, this enables clinicians to obtain reliable result within a few hours, thus facilitating the diagnostic workup. Rapid diagnosis is of high relevance for clinicians and patients, enabling start of targeted therapies to stop the immune activation underlying SJIA as early as possible.

### Supplementary Information

**Additional file 1: Supplemental Table 1.** Additional details of patient subgroups. **Supplemental Table 2.** Descriptive statistics of sCAL in different disease groups. **Supplemental Table 3. **Accuracy of sCAL turbo measurements in differentiating groups at higher cut-off levels. **Supplemental Table 4.** List of abbreviations.

**Additional file 2: Supplemental Fig. 1.** Correlation between sCAL turbo and ANC. Spearman’s p correlations were calculated in 147 samples with sCAL turbo and ANC (absolute neutrophil count per µl). **Supplemental Fig. 2.** Results of sCAL turbo measurements in ALL. Results of sCAL measurements in ALL patient groups divided into those with ANC <500/µl (*n*=81) or above (*n*=66) are shown using individual scatter plots, with red line showing median, error bars showing interquartile range (logarithmic scale of y-axis). ***p<0.001 (Mann-Whitney U test).

## Data Availability

All data generated or analyzed during this study are included in this published article (and its supplementary information files). The original datasets are available from the corresponding author on reasonable request.
