# Supplementary material for: A novel serum calprotectin (MRP8/14) particle-enhanced immuno-turbidimetric assay (sCAL turbo) helps to differentiate systemic juvenile idiopathic arthritis from other diseases in routine clinical laboratory settings
Source: Mol Cell Pediatr. 2023 Oct 25;10:14. doi: 10.1186/s40348-023-00168-0 (PMC10600080; doi:10.1186/s40348-023-00168-0)
Supplement: Supplementary file 1 — Additional file 1: Supplemental Table 1. Additional details of patient subgroups. Supplemental Table 2. Descriptive statistics of sCAL in different disease groups. Supplemental Table 3. Accuracy of sCAL turbo measurements in differentiating groups at higher cut-off levels. Supplemental Table 4. List of abbreviations. [file 40348_2023_168_MOESM1_ESM.docx]

**SUPPLEMENTAL TABLES**

**Supplemental Table 1. Additional details of patient subgroups.**

| **Infection group (n=51)** | | | | | | | |
| --- | --- | --- | --- | --- | --- | --- | --- |
|  | **n** | | **ESR, mm/h** | | **CRP, mg/dl** | **WBCs /µl** | **sCAL, ng/ml** |
| **All samples** | 51 | | 65  (5-109) | | 8.0  (1.2-44.1) | 11,375  (3,400-38,000) | 5,350 (250-18,500) |
| **Proven bacterial infections** | 19 | | 70 (18-109) | | 9.3 (1.7-44.1) | 12,500 (4,300-38,000) | 7,110 (550-14,600) |
| **Proven viral infections** | 7 | | 63 (32-86) | | 8.8 (1.2-20.4) | 7,000 (3,790-16,410) | 5,360 (2,550-18,500) |
| **Clinically diagnosed infections** | 25 | | 60 (5-100) | | 7.3 (1.2-41.0) | 10,400 (3,400-38,000) | 3,540 (250-17,270) |
| **SID group (n=126)** | | | | | | | |
|  | **n** | | **ESR, mm/h** | | **CRP, mg/dl** | **WBCs /µl** | **sCAL, ng/ml** |
| **All samples** | 126 | | 27  (1-140) | | 4.3  (1.0-32.8) | 9,520  (1,100-25,000) | 2,770 (270-19,290) |
| **Connective tissue diseases** | 5 | | 18 (7-90) | | 3.7 (1.1-14.0) | 6,950 (3,960-13,000) | 13,370  (1,210-19,290) |
| **Lymphoproliferative Disorder** | 3 | | 70 (8-132) | | 2.8 (2.1-32.0) | 11,900 (8,000-19,560) | 12,270  (2,660-14,640) |
| **Osteitis** | 4 | | 50 (6-53) | | 5.3 (1.0-8.0) | 5,450 (2,300-8,200) | 3,565  (640-4,050) |
| **Periodic fever syndromes** | 47 | | 25 (5-110) | | 2.1 (1.1-19.1) | 10,400 (1,560-24,200) | 2,450  (410-12,640) |
| **PFAPA** | 27 | | 29 (2-105) | | 9.1 (1.0-32.8) | 12,600 (5,000-25,000) | 3,530  (280-10,640) |
| **Unclear inflammation** | 30 | | 29 (1-132) | | 3.0 (1.0-22.8) | 9,700  (3,700-21,510) | 2,640  (560-10,170) |
| **Undifferentiated autoinflammation** | 5 | | 111 (82-140) | | 13.7 (3.2-20.1) | 6,800  (1,100-17,800) | 12,890  (2,690-16,510) |
| **Vasculitis** | 5 | | 14 (5-25) | | 1.5 (1.0-5.1) | 6,600 (1,400-10,000) | 2,240 (270-3,820) |
| **ALL group (n=147)** | | | | | | | |
| **Parameter** | **Arthralgia,**  **n (%)** | | **Fever,**  **n (%)** | | **LDH, U/l** | **Hb, g/dl** | **Platelets /µl** |
|  | 27 (18) | | 76 (52) | | 494  (126-5,000) | 8.0  (5.1-15.0) | 47,000  (2,000-376,000) |
| **nsJIA group (n=169)** | | | | | | | |
| **Category** | **Oligoarticular JIA** | | **Polyarticular**  **JIA** | | **Psoriatic**  **JIA** | **Enthesitis-related JIA** | **Undifferentiated JIA** |
| **Subcategory** | Pers. | Ext. | RF+ | RF- |  |  |  |
| **n** | 87 | 7 | 4 | 34 | 3 | 12 | 21 |
| *All data expressed as median (range) except otherwise stated.*  *n = number; ESR = erythrocyte sedimentation rate; CRP = C-reactive protein; WBC = white blood cells; sCAL = serum calprotectin; LDH = lactate dehydrogenase; Hb = hemoglobin; PFAPA = periodic fever with aphthous stomatitis, pharyngitis, and adenitis, ALL = acute lymphoblastic leukemia; SID = systemic inflammatory diseases; nsJIA = non-systemic juvenile idiopathic arthritis; Pers. = persistent; Ext. = extended; RF+ = rheumatic factor positive; RF- = rheumatic factor negative* | | | | | | | |

**Supplemental Table 2. Descriptive statistics of sCAL in different disease groups.**

*sCAL = serum calprotectin; SJIA = systemic juvenile idiopathic arthritis: nsJIA = non-systemic juvenile idiopathic arthritis; SIDs = systemic inflammatory diseases; ALL = acute lymphoblastic leukemia; CI = confidence interval; Std. = standard.*

**Supplemental Table 3. Accuracy of sCAL turbo measurements in differentiating groups at higher cut-off levels**

|  | ***SJIA vs all groups*** | ***SJIA vs infections*** | ***SJIA vs ALL*** |
| --- | --- | --- | --- |
| ***AUC (95%CI)*** | 0.960 (0.941-0.978) | 0.908 (0.862-0.953) | 0.992 (0.985-0.999) |
| ***Cut-Off (ng/ml)*** | 15,000 | 15,000 | 15,000 |
| ***Sensitivity (%)*** | 75 | 75 | 75 |
| ***Specificity (%)*** | 98 | 94 | 100 |
| ***Positive likelihood ratio (LR+)*** | 37.5 | 12.5 | $\infty$ |
| ***Negative likelihood ratio (LR-)*** | 0.26 | 0.27 | 0.25 |

*sCAL = serum calprotectin; SJIA = systemic juvenile idiopathic arthritis: nALL = acute lymphoblastic leukemia; AUC = Area under the curve; CI = confidence interval.*

**Supplemental Table 4. List of abbreviations**

| **Abbreviation** | **Explanation** |
| --- | --- |
| ALL | Acute lymphoblastic leukemia |
| ANC | Absolute neutrophil count |
| AUC | Area under curve |
| CI | Confidence Interval |
| CRP | C-reaktive protein |
| ELISA | Enzyme-linked immunosorbent assay |
| ESR | Erythrocyte sedimentation rate |
| FUO | Fever of unknown origin |
| IL | Interleukin |
| ILAR | International League of Associations for Rheumatology |
| LDH | Lactate dehydrogenase |
| LR- | Negative likelihood ratio |
| LR+ | Positive likelihood ratio |
| MIS-C | Multisystem inflammatory syndrome in children |
| MRP8/14 | Myeloid related proteins 8 and 14 |
| ng/ml | Nanogramm per milliliter |
| NLBB | NOPHO Leukemia BioBank |
| nsJIA | non-systemic JIA |
| PETIA | Particle enhanced immuno-turbidimetric assay |
| ROC | Receiver operating characteristic |
| sCAL | Serum-calprotectin |
| SIDs | Systemic inflammatory diseases |
| SJIA | Systemic juvenile idiopathic arthritis |
| SJIA-LD | SJIA-associated lung disease |
| SJIA-MAS | Macrophage activation syndrome |
| SPSS | Statistical package for social sciences |
| WBC | White blood cells |
