# Supplementary material for: A novel serum calprotectin (MRP8/14) particle-enhanced immuno-turbidimetric assay (sCAL turbo) helps to differentiate systemic juvenile idiopathic arthritis from other diseases in routine clinical laboratory settings
Source: Mol Cell Pediatr. 2023 Oct 25;10:14. doi: 10.1186/s40348-023-00168-0 (PMC10600080; doi:10.1186/s40348-023-00168-0)
Supplement: Supplementary file 2 — Additional file 2: Supplemental Fig. 1. Correlation between sCAL turbo and ANC. Spearman’s p correlations were calculated in 147 samples with sCAL turbo and ANC (absolute neutrophil count per µl). Supplemental Fig. 2. Results of sCAL turbo measurements in ALL. Results of sCAL measurements in ALL patient groups divided into those with ANC <500/µl (n=81) or above (n=66) are shown using individual scatter plots, with red line showing median, error bars showing interquartile range (logarithmic scale of y-axis). ***p<0.001 (Mann-Whitney U test). [file 40348_2023_168_MOESM2_ESM.docx]

**SUPPLEMENTAL FIGURES**

**Supplemental Figure 1. Correlation between sCAL turbo and ANC**

Spearman’s p correlations were calculated in 147 samples with sCAL turbo and ANC (absolute neutrophil count per µl).

**Supplemental Figure 2. Results of sCAL turbo measurements in ALL**

Results of sCAL measurements in ALL patient groups divided into those with ANC <500/µl (n=81) or above (n=66) are shown using individual scatter plots, with red line showing median, error bars showing interquartile range (logarithmic scale of y-axis). ****p<0.001* (Mann-Whitney U test).
